# Anti-interleukin-6 therapy through application of a monogenic protein inhibitor *via* gene delivery

**DOI:** 10.1038/srep14685

**Published:** 2015-10-01

**Authors:** Dieter Görtz, Gerald S. Braun, Yuichi Maruta, Sonja Djudjaj, Claudia R. van Roeyen, Ina V. Martin, Andrea Küster, Hildegard Schmitz-Van de Leur, Jürgen Scheller, Tammo Ostendorf, Jürgen Floege, Gerhard Müller-Newen

**Affiliations:** 1 Institute of Biochemistry and Molecular Biology, RWTH Aachen University, Aachen, Germany; 2 Division of Nephrology and Immunology, RWTH Aachen University, Aachen, Germany; 3 Institute of Pathology, RWTH Aachen University, Aachen, Germany; 4 Institute of Biochemistry and Molecular Biology II, Medical Faculty, Heinrich-Heine-University, Düsseldorf, Germany

## Abstract

Anti-cytokine therapies have substantially improved the treatment of inflammatory and autoimmune diseases. Cytokine-targeting drugs are usually biologics such as antibodies or other engineered proteins. Production of biologics, however, is complex and intricate and therefore expensive which might limit therapeutic application. To overcome this limitation we developed a strategy that involves the design of an optimized, monogenic cytokine inhibitor and the protein producing capacity of the host. Here, we engineered and characterized a receptor fusion protein, mIL-6-RFP-Fc, for the inhibition of interleukin-6 (IL-6), a well-established target in anti-cytokine therapy. Upon application in mice mIL-6-RFP-Fc inhibited IL-6-induced activation of the transcription factor STAT3 and ERK1/2 kinases in liver and kidney. mIL-6-RFP-Fc is encoded by a single gene and therefore most relevant for gene transfer approaches. Gene transfer through hydrodynamic plasmid delivery in mice resulted in hepatic production and secretion of mIL-6-RFP-Fc into the blood in considerable amounts, blocked hepatic acute phase protein synthesis and improved kidney function in an ischemia and reperfusion injury model. Our study establishes receptor fusion proteins as promising agents in anti-cytokine therapies through gene therapeutic approaches for future targeted and cost-effective treatments. The strategy described here is applicable for many cytokines involved in inflammatory and other diseases.

Anti-cytokine therapy is a significant step forward in the current treatment of immunologic and other inflammatory diseases and has therapeutic potential in cancer. Anti-inflammatory biologics in clinical use today are mainly engineered antibodies which block cytokines such as TNF, IL-6 and IL-1 or one of their receptors^1^,^2^,^3^,^4^,^5^. Engineered soluble cytokine-binding receptors are an effective alternative to antibodies offering easier production, better suitability for gene therapeutic delivery and therefore add to the available choice of therapeutics. Surprisingly, far fewer substances of this class have been developed to clinical maturity^6^.

Most cytokines signal through heteromeric receptors and often two or more different receptor chains contribute to high-affinity binding of the ligand. In the case of IL-6, the *α*-receptor subunit in its soluble form (sIL-6R*α*) is an agonistic molecule that in combination with IL-6 stimulates cells expressing the *β*-subunit gp130^7^. A soluble form of gp130 (sgp130), in turn, potently antagonizes the complex of IL-6 and sIL-6R*α*^8^,^9^. Based on these observations, we and others developed a strategy to block IL-6 with a fusion protein consisting of the ligand-binding domains of IL-6R*α* and gp130^10^,^11^. These receptor fusion proteins (RFPs) or cytokine traps turned out to be potent and specific inhibitors of cytokine activity. Meanwhile, this strategy has been successfully applied *in vitro* for the inhibition of leukemia inhibitory factor (LIF)^12^, oncostatin M (OSM)^13^, IL-31^14^ and other cytokines^6^. Since a RFP is encoded by a single gene, RFPs are well suited for gene therapeutic approaches where the biological is endogenously expressed instead of externally applied. Gene therapeutic application of an antibody comprising a heavy and a light chain may be more difficult to achieve given the requirement of two encoding genes.

We previously described a murine IL-6-RFP as a potent inhibitor of both classical (through membrane-bound IL-6R*α*) and IL-6 trans-signaling (through sIL-6R*α*) with an inhibitory activity comparable to that of an IL-6 neutralizing antibody^15^. Here we show that an engineered murine IL-6 receptor fusion protein, mIL-6-RFP-Fc, optimized for expression in mice and subsequent detection in body fluids and tissues, is expressed upon gene transfer in mice, blocks IL-6 activity *in vivo* and alleviates ischemia-reperfusion injury of the kidney.

## Results

### Generation, optimization and expression of mIL-6-RFP-Fc

**Figure 1:**
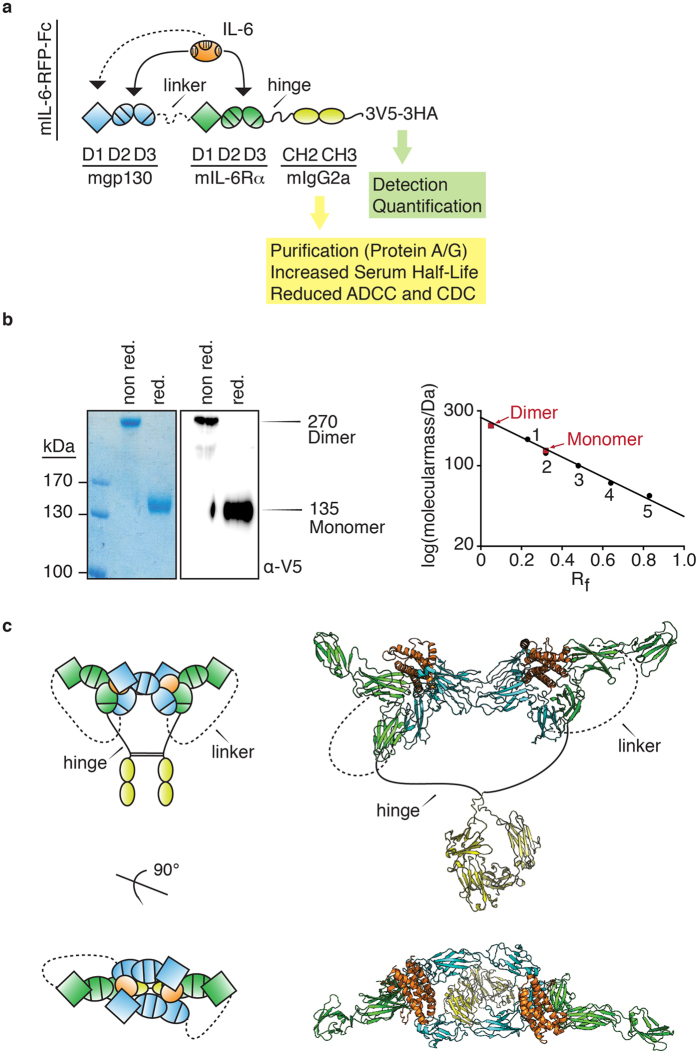
Design, characterization and structural model of mIL-6-RFP-Fc. (**a**) mIL-6-RFP-Fc consists of domains (D) D1-D3 of murine gp130 connected through a flexible linker with D1-D3 of murine IL-6R*α* for high-affinity binding of murine IL-6, the Fc-fragment (CH2-CH3) including the hinge region of mIgG2a which has been mutated to reduce antibody-dependent cellular cytotoxicity (ADCC) and complement-dependent cytotoxicity (CDC)^50^ followed by three V5 and three HA epitopes (3V5-3HA) for sensitive detection by immunofluorescence and reliable quantification by ELISA. Arrows indicate binding of IL-6 to mIL-6-RFP-Fc with the dashed line indicating binding to a second mIL-6-RFP-Fc resulting in the formation of a complex in analogy to the hexameric IL-6 receptor complex (shown in (**c**)). Secretion of mIL-6-RFP-Fc is driven by the signal sequence of preprotrypsin (not shown) as established previously for mIL-6RFP^15^. (**b**) Purified mIL-6-RFP-Fc was analyzed by 7.5% SDS/PAGE. Purity and identity was determined by staining with Coomassie brilliant blue and Western blotting, respectively, under non-reducing and reducing conditions. The molecular mass of mIL-6-RFP-Fc was determined by calculating the R_f_ of five marker proteins and inserting the R_f_ of mIL-6-RFP-Fc monomer and dimer into the equation for the linear regression. (**c**) Schematic representation (left panel) and structural model (right panel) of the (IL-6)_2_(mIL-6-RFP-Fc)_2_ inhibitory complex. The structural model is based on the crystal structures of the human hexameric IL-6 signaling complex (PDB: 1P9M), human IL-6R*α* (PDB: 1N26), and the hinge region, CH2 domain and CH3 domain of murine IgG2A (PDB: 1IGT).

mIL-6-RFP-Fc was generated by adding an engineered mIgG2a Fc-fragment followed by a transferable tag for detection and quantification to the C-terminus of our previously reported receptor fusion protein mIL-6-RFP for the inhibition of human, rat and murine IL-6^15^ (Fig. 1a).

The Fc-fragment facilitates purification and is expected to increase serum half-life of the recombinant protein. Moreover, the Fc-fragment enforces dimerization of the fusion protein through disulfide bond formation as shown by SDS-PAGE under non-reducing and reducing conditions (Fig. 1b, left panel) increasing its avidity towards IL-6. From SDS-PAGE apparent molecular masses of 270 kDa for the dimer and 135 kDa for the monomer have been calculated (Fig. 1b, right panel). The dimer is perfectly tailored for adapting the conformation of the hexameric IL-6 receptor complex required for high-affinity IL-6-binding^16^ (Fig. 1c).

**Figure 2:**
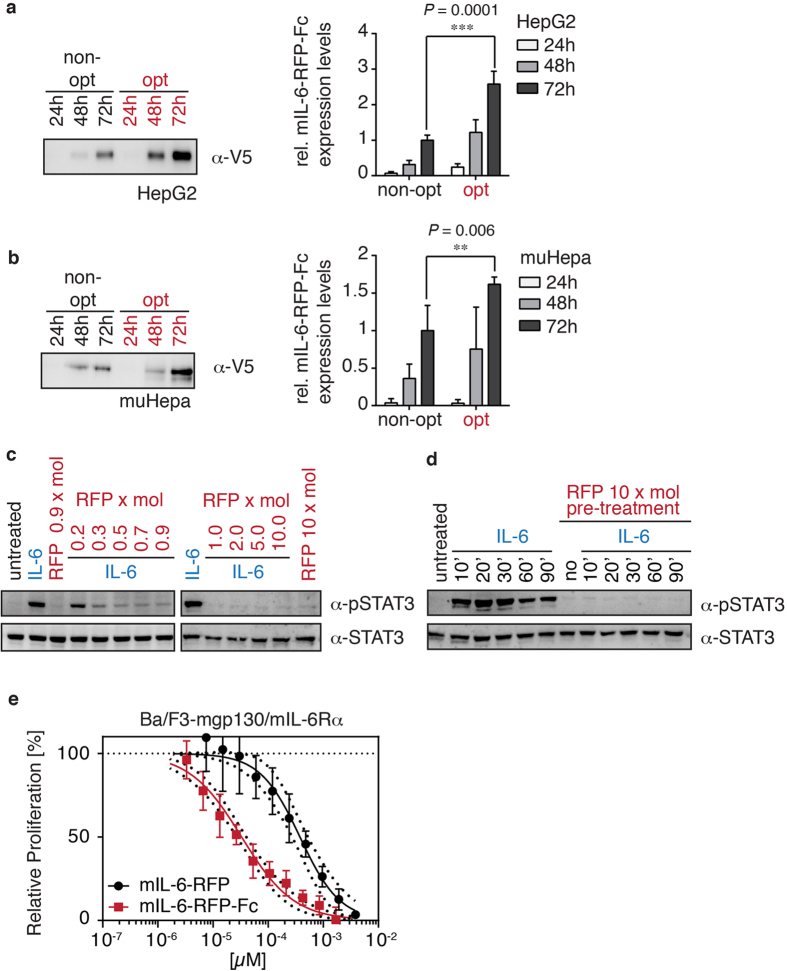
Expression of gene-optimized mIL-6-RFP-Fc and inhibitory activity in vitro. Western blot analysis of equal volumes of conditioned media harvested from (**a**) HepG2 and (**b**) muHepa cells transiently transfected with optimized (opt) or non-optimized (non-opt) mIL-6-RFP-Fc expression plasmids after the indicated time points using an antibody directed against the V5-epitope (*α*-V5).Bar charts show relative expression of mIL-6-RFP-Fc from densitometric analyses of Western blots. For comparison, densitometric levels from the secretion of non-opt mIL-6-RFP-Fc at 72 h were set to 1. Results of five independent experiments are presented (means ± SD, **p ≤ 0.01, ***p ≤ 0.001 one-sided t-test). (**c**) muHepa cells were stimulated for 20 min with IL-6 (20 ng/ml), mIL-6-RFP-Fc (RFP) or IL-6 (20 ng/ml) preincubated with mIL-6-RFP-Fc (RFP) at concentrations ranging from 22.25 to 1335 ng/ml for 15 min corresponding to molar ratios of mIL-6-RFP-Fc:IL-6 as indicated (x mol). Cellular lysates were analyzed by Western blotting for phosphorylation of STAT3 at Y705 (*α*-pSTAT3) and total STAT3 (*α*-STAT3) as a loading control. (**d**) muHepa cells were pretreated for 120 min with a 10-fold molar excess of mIL-6-RFP-Fc (1335 ng/ml) and subsequently stimulated with IL-6 (20 ng/ml) for 20 min. Phosphorylation of STAT3 was analyzed as described in (**c**). (**e**) Equal numbers of Ba/F3-mgp130/mIL-6R*α* cells were incubated with a constant amount of IL-6 (37 pM) and serial 2-fold dilutions of either mIL-6-RFP-Fc (starting concentration 3.9 nM) or mIL-6-RFP (starting concentration 3.4 nM). After 60 h of incubation, viable cells were quantified using a colorimetric XTT assay. Data shown are means ± SD (n = 6).

The cDNA encoding mIL-6-RFP-Fc was optimized for gene delivery in mice by replacing 32% of the codons for those being preferred in mice. In addition, potential internal inhibitory motifs, AT-rich instability motifs, repeat sequences, internal polyadenylation sites, as well as splice donor and acceptor sites were eliminated. Human (HepG2) and murine hepatocarcinoma (muHepa) cells were transiently transfected with vectors expressing the original or optimized cDNA encoding mIL-6-RFP-Fc under the control of the CMV promoter. Over time increasing amounts of the recombinant protein were detected in the supernatant. Compared to the original cDNA the optimized cDNA yielded an about 2.5-fold and 1.6-fold increase in protein production in HepG2 and muHepa cells, respectively (Fig. 2a,b). With the optimized construct stably transfected HEK293 cells were generated for continuous protein production. mIL-6-RFP-Fc was isolated from conditioned media through affinity chromatography with protein A Sepharose (see purified protein in Fig. 1b) yielding about 4 mg of purified protein per liter of conditioned medium.

### Effective inhibition of IL-6 in vitro and in vivo with recombinant mIL-6-RFP-Fc

To characterize the bioactivity of mIL-6-RFP-Fc, muHepa cells were stimulated with murine IL-6 that had been preincubated with purified mIL-6-RFP-Fc in molar ratios ranging from 0.2 to 10. As a read-out of IL-6 activity tyrosine phosphorylation of STAT3 was analyzed (Fig. 2c). Complete inhibition of IL-6 was already achieved at about equimolar concentrations of mIL-6-RFP-Fc and IL-6. In the next experiment, mIL-6-RFP-Fc and IL-6 were not preincubated, but cells were pretreated with mIL-6-RFP-Fc (Fig. 2d). Subsequent addition of IL-6 did not elicit phosphorylation of STAT3 over a prolonged period of time indicating that even in direct competition with the cell surface receptors mIL-6-RFP-Fc neutralizes IL-6 immediately and completely. Activities of the original mIL-6-RFP^15^ and the novel mIL-6-RFP-Fc were compared on Ba/F3 cells stably expressing mIL-6R*α* and mgp130 (Ba/F3-mgp130/mIL-6R*α*). IL-6-dependent proliferation of Ba/F3-mgp130/mIL-6R*α* cells was inhibited in a concentration-dependent manner by both inhibitors. However, mIL-6-RFP-Fc’s inhibitory activity was one order of magnitude higher compared to the non-optimized version of mIL-6-RFP (Fig. 2e; IC50 mIL-6-RFP-Fc: 3.3 × 10^−5^ μM, IC50 mIL-6-RFP: 3.8 × 10^−4^ μM) which is in agreement with the higher avidity of mIL-6-RFP-Fc as proposed in our model (Fig. 1c) and with the increase of the activity of the IL-6 trans-signaling inhibitor sgp130 upon Fc fusion^17^.

**Figure 3:**
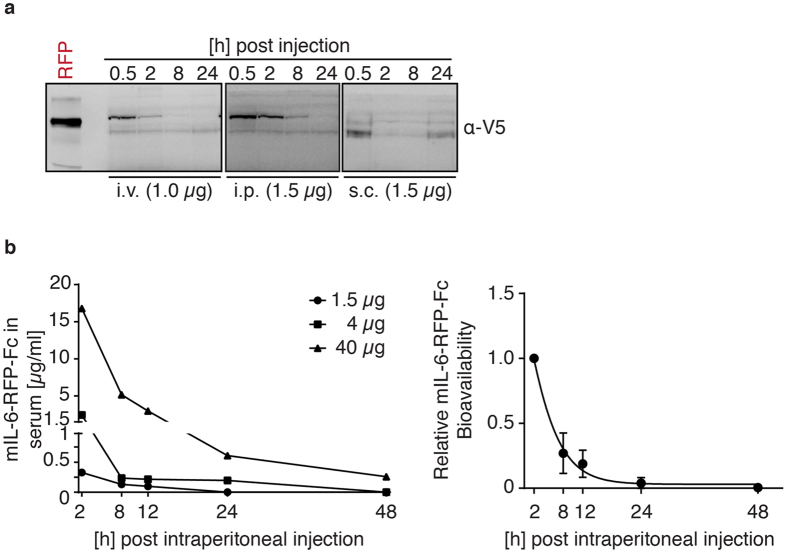
Pharmacokinetic properties of mIL-6-RFP-Fc. (**a**) Western blot analysis of mIL-6-RFP-Fc in mouse serum samples (2 *μ*l serum/lane) obtained at the indicated time points following administration of indicated amounts of mIL-6-RFP-Fc either intravenously (i.v.), intraperitoneally (i.p.) or subcutaneously (s.c.) using an antibody directed against the V5-epitope (*α*-V5). (**b**) Left: mIL-6-RFP-Fc serum concentrations over time following intraperitoneal administration of the indicated amounts of recombinant protein as determined by an ELISA based on capture and detection of the HA- and V5-epitopes, respectively. Depicted are individual animals. Right: Statistical evaluation by normalization of each animals’ values on its 2 h serum level. Calculated time constant: t_1/2_ = 3.5 h.

To establish its bioavailability in a mammalian system, mIL-6-RFP-Fc was administered to mice as 1 *μ*g intravenously (i.v.), 1.5 *μ*g intraperitoneally (i.p.) and 1.5 *μ*g subcutaneously (s.c.) and recovered via blood sampling. For i.v. injection a lower amount was chosen because a higher recovery was expected upon direct administration to the blood stream. As determined by Western blot analysis of serum samples, the i.v. and i.p. routes gave rise to the highest systemic levels with the latter resulting in prolonged serum detectability with no signs of major degradation of the fusion protein (Fig. 3a). S.c. application did not result in significant plasma levels, which might be explained by the known slow-onset and prolonged pharmacokinetics of this route. It should be noted that a non-optimized, non-Fc-containing variant of mIL-6-RFP^15^ was successfully applied s.c. for local IL-6 antagonism in a cutaneous tumor model^18^. To further characterize the pharmacokinetics of mIL-6-RFP-Fc, different amounts, i.e. 1.5, 4, and 40 *μ*g were injected i.p., followed by repeated blood sampling for quantitative serum ELISA directed against the epitope tags. At the highest amount (40 *μ*g) applied in one single administration, mIL-6-RFP-Fc could be detected in serum for up to 48 h (Fig. 3b, left diagram). Normalization of the different animals’ serum mIL-6-RFP-Fc levels on their respective 2 h value, allowed for statistical analysis and determination of a time constant (t_1/2_ = 3.5 h) across the amounts of protein spiked (Fig. 3b, right diagram). The observed half-life is identical with the initial clearance determined for an IL-2-Fc fusion protein harbouring the same Fc-fragment (mIgG2a)^19^.

**Figure 4:**
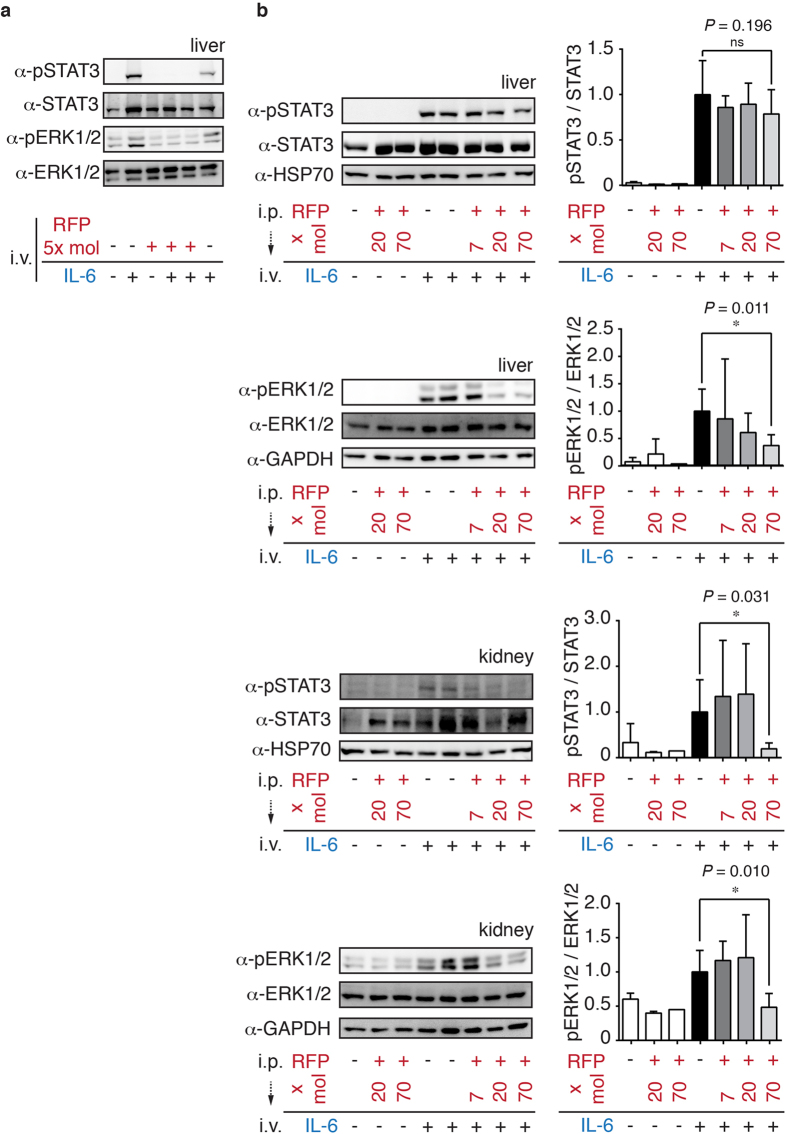
Inhibition of IL-6 dependent STAT3 and ERK phosphorylation by mIL-6-RFP-Fc *in vivo*. (**a**) Western blot analysis of mouse liver lysates prepared 15 min following intravenous administration of either PBS, IL-6 (100 ng) or preincubated IL-6/mIL-6-RFP-Fc-complexes (100 ng/3.5 *μ*g, corresponding to a molar ratio of 1:5, incubation for 30 min). (**b**) Representative Western blot of mouse liver and kidney lysates prepared 15 min following intravenous administration of either PBS or IL-6 (100 ng). Mice were pretreated for 45 min with PBS or mIL-6-RFP-Fc i.p. at dosages of 4, 12 or 40 *μ*g, corresponding to molar mIL-6-RFP-Fc (RFP): IL-6 ratios as indicated. Phosphorylated STAT3 (*α*-pSTAT3), phosphorylated ERK1/2 (*α*-pERK1/2), total STAT3 (*α*-STAT3) and total ERK1/2 (*α*-ERK1/2) were detected. GAPDH and HSP70 served as loading controls. Statistical evaluation of experiments are depicted as bar charts. Data shown are means ± SD, *p ≤ 0.05, one-sided t-test (n = 2 for PBS i.v.-stimulated groups; n = 5 for PBS i.p./IL-6 i.v.; n = 3 for RFP 7x i.p./IL-6 i.v.; n = 3 for RFP 20x i.p./IL-6 i.v.; n = 4 for RFP 70x i.p./IL-6 i.v.). Because of variations in STAT3 expression levels, pSTAT3 was normalized relative to total STAT3. Similarly, pERK1/2 was normalized relative to total ERK1/2.

To determine the efficacy of mIL-6-RFP-Fc in mice, we established a model of IL-6-stimulated STAT3 and ERK1/2 phosphorylation in liver and kidney tissues. In analogy to the *in vitro* experiments, mIL-6-RFP-Fc was either pre-incubated with IL-6 (Fig. 4a) or the proteins were administered sequentially (Fig. 4b). In both scenarios mIL-6-RFP-Fc significantly reduced IL-6 downstream signaling. Pretreatment with 40 *μ*g (70× molar excess) of mIL-6-RFP-Fc i.p. for 45 min attenuated STAT3 and ERK1/2 phosphorylation by 12 and 63% in the liver and by 80 and 50% in the kidney, respectively. Phosphorylation was assessed by the ratio of phosphorylated versus total STAT3 or ERK1/2 (Fig. 4b). Variations of total STAT3 levels might be due to the fact that activated STAT3 induces its own gene expression^20^,^21^. Collectively, these data established mIL-6-RFP-Fc as a potent therapeutic agent for the inhibition of IL-6 *in vitro* and *in vivo*.

### Effective gene delivery and inhibitory capacity of mIL-6-RFP-Fc in mice and use in a preclinical model of acute kidney injury

Anti-cytokine therapy through application of recombinant protein has been shown to be effective but protein production is expensive and tedious. We argued that a gene therapeutic approach, where small amounts of a delivered plasmid generate substantial protein levels by the recipient himself, might be much more efficient. mIL-6-RFP-Fc is encoded by a single gene and therefore ideally suited for gene delivery.

**Figure 5:**
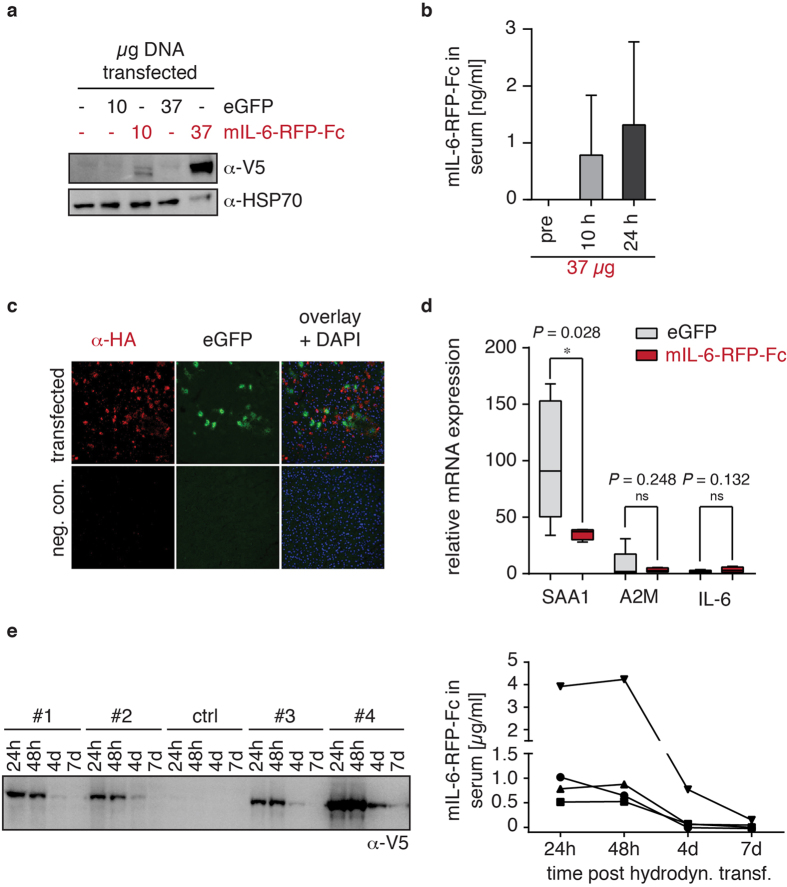
Expression of mIL-6-RFP-Fc upon gene transfer through hydrodynamic transfection. (**a**) Western blot analysis of liver lysates from mice hydrodynamically transfected with control vector (GFP) lacking the epitopes or mIL-6-RFP-Fc using an antibody directed against the V5-epitope of mIL-6-RFP-Fc (*α*-V5). Amounts of plasmid transfected as indicated. Mice were sacrificed 24 h post transfection. As a negative control, a mouse treated with transfection agent without plasmid is shown. Detection of HSP70 served as a loading control (*α*-HSP70). (**b**) mIL-6-RFP-Fc serum concentrations as determined by ELISA at time points indicated. n = 4 animals; subsequently also analyzed in (**d**). (**c**) Immunofluorescence of liver cryosections 24 h post hydrodynamic gene delivery. Co-transfection with expression vectors encoding mIL-6-RFP-Fc (as detected by *α*-HA, red) and GFP (green) was performed. As a negative control, a mouse treated with transfection agent without plasmid is shown. (**d**) qRT-PCR of liver acute phase mRNAs 24 h after hydrodynamic delivery of 37 *μ*g of either control plasmid or mIL-6-RFP-Fc-encoding plasmid. SAA1, serum amyloid A; A2M, *α*2-macroglobulin. Data were normalized and calculated using the housekeeper GAPDH and the ΔΔCT method^51^. X-fold expression relative to mean of healthy normal mice. n = 4 (mIL-6-RFP-Fc) and n = 5 (GFP) animals/group. Box plots, whiskers indicating minimal to maximal values, *p ≤ 0.05, one-sided t-test. (**e**) Serum levels of mIL-6-RFP-Fc from 4 additional mice (#1–#4) over time following hydrodynamic transfection with 37 *μ*g plasmid. Western blot using an antibody directed against the V5-epitope (*α*-V5, left panel) and corresponding quantification by ELISA (right panel). ctrl, mouse transfected with empty vector.

In order to achieve high systemic levels of circulating mIL-6-RFP-Fc we used the method of hydrodynamic plasmid delivery *via* the tail vein, which mainly results in transfection of the liver and to a much lower extent of the kidney and other organs (1:100–1000)^22^. The phosphoenolpyruvate carboxykinase (PEPCK) promoter was chosen because it is active in the liver and generates high systemic levels of soluble proteins^23^,^24^. Following gene delivery, mIL-6-RFP-Fc was detected in liver tissue (Fig. 5a) and serum (Fig. 5b). The concentration of expressed protein in the liver correlated well with the amount of plasmid used for transfection (i.e. 10 and 37 *μ*g/animal, respectively). Using the latter amount, a mIL-6-RFP-Fc serum level at 24 hrs of 1.3 ± 1.5 *μ*g/ml (mean ± SD; n = 4) was achieved, thus corresponding to the 2 h and 12–24 h levels upon singular i.p. spiking of 4 and 40 *μ*g of mIL-6-RFP-Fc, respectively (Fig. 3b). Subsequently, 37 *μ*g of plasmid/animal were used for hydrodynamic transfection throughout all experiments. Analysis of liver sections of mice that were simultaneously transfected with plasmid encoding mIL-6-RFP-Fc and GFP revealed that a single cell either expresses the fusion protein or GFP but rarely both (Fig. 5c). This finding emphasizes the advantage of a single gene construct such as receptor fusion proteins over proteins encoded by multiple genes such as antibodies for gene transfer approaches. The bioactivity of mIL-6-RFP-Fc administered through gene transfer could be directly confirmed by the suppression of hepatic acute phase protein production which had been induced by the invasive transfection procedure. Expression of mRNA for serum amyloid A1 (SAA1) was significantly reduced and expression of mRNA for *α*2-macroglobulin (A2M) showed a trend of a reduction (Fig. 5d). Both proteins are known to be governed by IL-6^25^. A trend for slightly increased IL-6 mRNA levels in the mIL-6-RFP-Fc treated group might reflect compensatory IL-6 production (Fig. 5d). The fact that SAA1 is not completely suppressed in this model might be potentially explained by other SAA1-inducing cytokines such as IL-1 and TNF being released following hydrodynamic transfection.

To assess the usefulness of the hydrodynamic transfection approach for disease models, the pharmacokinetics of our RFP were evaluated in more detail. mIL-6-RFP-Fc was detected in serum for up to one week after gene transfer, again with no signs of degradation. Average serum levels of 1.6 *μ*g/ml (±1.6 SD; n = 4) mIL-6-RFP-Fc were achieved for at least 48 h (Fig. 5e), indicating continuous synthesis as opposed to singular spike and turnover (Fig. 3b).

**Figure 6:**
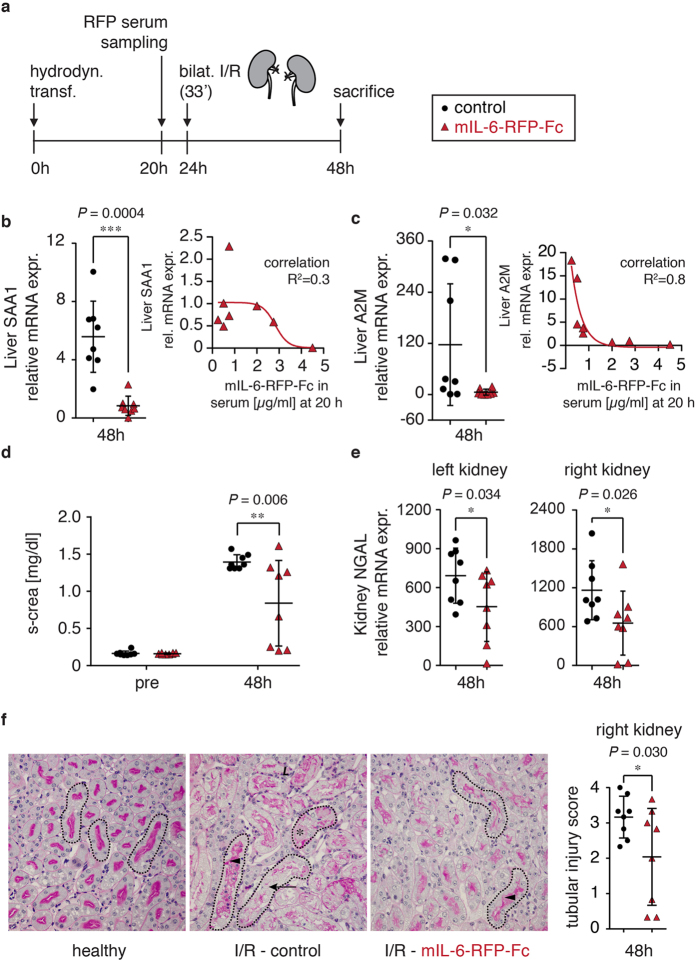
Inhibition of IL-6 through gene transfer of mIL-6-RFP-Fc in a renal ischemia-reperfusion model. (**a**) Schematic of experimental design. Bilateral (bilat.) renal ischemia-reperfusion (I/R, 33 min of ischemia) performed 24 h following hydrodynamic transfection (hydrodyn. transf.) of 37 *μ*g mIL-6-RFP-Fc or 37 μg empty vector. Serum levels of mIL-6-RFP-Fc were measured at 20 h post hydrodynamic transfection. Serum creatinine was assessed 5 days prior to gene delivery and at sacrifice. All data are from n = 8 animals/group, shown as means (horizontal bars) ± SD (vertical bars) and single values (symbols), *p ≤ 0.05, **p ≤ 0.01, ***p ≤ 0.001 one-sided t-test. (**b**,**c**) qRT-PCR of acute phase mRNAs from liver of sacrificed mice as calculated relative (x-fold) to mean of healthy control mice using housekeeper GAPDH and the ΔΔCT method^51^. Expression of both acute phase proteins by mIL-6-RFP-Fc transfection is significantly reduced (red triangles) in comparison to control vector (black dots). Nonlinear regression analysis revealed that suppression of SAA1 and A2M correlates with serum mIL-6-RFP-Fc levels. (**d**) Increased serum creatinine following I/R is significantly reduced by prior transfection with mIL-6-RFP-Fc. Verification of t-test results by ANOVA with Tuckey multiple group analysis revealed an overall p ≤ 0.0001 and an identical significance level of  ≤ 0.01 for I/R control versus mIL-6-RFP-Fc. (**e**) Transcription of the tubular injury marker NGAL in both kidneys as assessed using housekeeper GAPDH and the ΔΔCT method^51^, indicating the significant mitigation of renal injury upon hydrodynamic transfection of mIL-6-RFP-Fc (red triangles) compared to empty vector (black dots). (**f**) Representative renal tissue sections, original magnifications, ×200. Dotted lines, representative renal tubules. Normal healthy kidney showing homogeneous basophilic and eosinophilic staining of the cytoplasm and of the apical brush border membrane, respectively. Following I/R, tubular dilatation/atrophy (arrowheads), loss of the brush border (arrow), tubular necrosis (asterisk) and leukocyte infiltration (L) ensue, the degree of which is mitigated following transfection with mIL-6-RFP-Fc. Far right: statistical analysis of tissue injury score for the right kidney (left kidney not assessed). Red triangles, mIL-6-RFP-Fc; black dots, empty vector. See methods for details.

To assess the therapeutic potential of mIL-6-RFP-Fc produced *via* gene delivery in a relevant disease model we applied a model of acute kidney injury (AKI) by transient bilateral renal ischemia followed by reperfusion for 24 hours (I/R). This well-established model of tubular cell necrosis^26^ has been previously demonstrated to depend critically on IL-6^27^. Injury is significantly reduced in IL-6^−/−^ mice and can also be alleviated by the adoptive transfer of IL-6^−/−^ bone marrow or by treatment with IL-6 neutralizing antibodies, resulting in improved renal serum and histological parameters^28^,^29^. Our experimental design is outlined in Fig. 6a. Mice with detectable mIL-6-RFP-Fc serum levels (0.25–4.5 *μ*g/ml; mean 1.5 *μ*g/ml ± SD 1.5) at 20 h post hydrodynamic transfection or equally treated mice having received control vector were subjected to bilateral ischemia of 33 minutes duration at 24 h and sacrificed at 48 h for further organ analysis. Successful transfection and biological activity of mIL-6-RFP-Fc was confirmed by suppressed hepatic SAA1 and A2M mRNAs, which also correlated with the 20 h serum level of mIL-6-RFP-Fc (Fig. 6b,c). In line with the previous studies^28^,^29^, we observed similar beneficial effects on renal endpoints. Serum creatinine, intrarenal mRNA synthesis of the acute kidney injury marker neutrophil gelatinase-associated lipocalin (NGAL)/lipocalin-2 (Lcn2)^30^,^31^ and histologic tubular injury scoring were significantly reduced following transfection with mIL-6-RFP-Fc (Fig. 6d–f). These data are also in line with another report in which IL-6^−/−^ mice were protected in the initial phase of a mercury-induced proximal tubular injury model^32^. It must be noted that the renal functional and renal histologic endpoints of the mIL-6-RFP-Fc group exhibited a comparatively wide variance (Fig. 6d,f). This is in agreement with published data on this complex acute kidney injury model^28^ (see Discussion section for details). By combining the data from the hydrodynamic transfections of 16 individual animals described herein (Fig. 5b n = 4, Fig. 5e n = 4, Fig. 6 n = 8), a very consistent mean serum expression level of mIL-6-RFP-Fc within the first 20–48 h of 1.5 *μ*g/ml; ± SD 1.4 can be inferred.

Collectively, the consistent reduction of the hepatic acute phase response in both the hepatic transfection and the I/R model (Figs 5 and 6, respectively) and the significant – though variant – benefit of renal endpoints in I/R (Fig. 6) establish endogenous expression of mIL-6-RFP-Fc *via* gene transfer as an effective treatment in murine disease models.

## Discussion

There is an ever-growing requirement for biologics to treat inflammatory diseases and cancer. Production of biologics involves expression of recombinant proteins in cell culture and subsequent purification under standardized conditions. The entire process is time-consuming, intricate and costly. Consequently, increasing prescription rates of biologics will become a heavy burden for health care systems^33^. Therefore, alternative cost-effective strategies that circumvent recombinant protein production are highly desired. A potential solution of the problem is to use the protein-producing capability of the host and to deliver the genetic information instead of the protein itself. This strategy of therapeutic gene delivery would work best for biologics encoded by a single gene and requires optimization of the protein, the encoding cDNA and reliable gene transfer methods.

Here, we have introduced mIL-6-RFP-Fc as a receptor fusion protein for the inhibition of IL-6 that has been optimized for application through gene delivery in mice. IL-6 is a well-established therapeutic target in rheumatic disease, other inflammatory diseases and cancer^34^,^35^,^36^,^37^,^38^. Starting with our previously described mIL-6-RFP^15^, we added an Fc-fragment for several purposes. Cysteine residues within the Fc-fragment enforce dimerization of mIL-6-RFP-Fc through disulfide bond formation favouring the formation of an inhibitory complex consisting of a mIL-6-RFP-Fc dimer that binds two IL-6 molecules. This stoichiometry is analogous to the high-affinity hexameric receptor complex consisting of two molecules of each IL-6, sIL-6R*α* and gp130 that has been verified by X-ray crystallography^16^. Earlier work demonstrated that IL-6-RFP indeed forms complexes similar to the native IL-6 receptor complex^39^. The extraordinary inhibitory potency of mIL-6-RFP-Fc is demonstrated by its capacity to inhibit IL-6-mediated proliferation of Ba/F3-mgp130/mIL-6R*α* cells with a tenfold higher potency than the original mIL-6-RFP.

Furthermore, the Fc-fragment of IgG interacts with the neonatal Fc receptor (FcRn) that mediates recycling of pinocytosed protein, thus increasing the plasma half-life of the fusion protein^40^. Plasma half-life can be further increased through mutations within the Fc-fragment that affect the Fc-FcRn interaction^41^. Finally, when mIL-6-RFP-Fc is produced as a recombinant protein the Fc-fragment allows convenient purification through affinity chromatography with immobilized Protein A or G. mIL-6-RFP-Fc can be reliably quantified with an ELISA directed against the two triple tags (3V5-3HA) that follow the Fc-fragment. By simple cloning procedures, the Fc-3V5-3HA module can be easily transferred to other receptor fusion proteins so that different proteins can be quantified by the same assay.

Interestingly, the expression level of mIL-6-RFP-Fc increased through optimization of the cDNA sequence that affects codon usage and all sequences that could interfere with stability and translation of the mRNA. An analogous human hIL-6-RFP-Fc for clinical trials should therefore be optimized the same way. A great advantage of an endogenously produced therapeutic protein is that the glycosylation is entirely host-like which cannot be achieved by recombinant protein production. This feature is expected to dramatically reduce immunogenicity which often is a result of aberrant glycosylation^42^.

The experimental method of hydrodynamic transfection^22^ allowed us to effectively characterize the biological action of mIL-6-RFP-Fc *in vivo* but obviously this mode of gene delivery will not be suited for the treatment of humans. For future therapeutic applications in patients, an analogous hIL-6-RFP-Fc designed for delivery through safe virus-based transfection methods such as integrating lentiviral vectors or non-integrating adeno-associated virus-derived vectors will be required and is under current development^43^. Similarly, transfer vectors can be designed that control expression of mIL-6-RFP-Fc through organ- or tissue-specific promoters so that the consequences of local IL-6 inhibition can be studied.

A large variance within the IL-6 knockout groups in the I/R model was also observed in another study (at least twofold of the wildtype control groups)^28^. To better understand this phenomenon a careful correlation analysis of the 20 h serum mIL-6-RFP-Fc levels and the associated renal outcomes was undertaken. While the animal with the highest serum level of 4.5 *μ*g/ml exhibited the lowest serum creatinine level (0.21 mg/dl) and the lowest average NGAL expression level (right kidney: 40-fold, left kidney 156-fold of normal) no clear correlation between these outcome markers and the mIL-6-RFP-Fc level was found in the other animals (data not shown). On the other hand, a clear dose-response effect was observed in the inhibition of the hepatic acute phase response (Fig. 6b,c).

Intriguingly, in the hepatic stress response at 24 h following hydrodynamics based transfection (Fig. 5d) acute phase transcriptional suppression by mIL-6-RFP-Fc is significant but incomplete while in the I/R model it is total with some animals ranging below the level of healthy controls (Fig. 6b,c). Indeed, as discussed above, 24 h post hydrodynamics stress non-IL-6 driven SAA1 transcription (IL-1, TNF) might be the case, while for the I/R model it has been elegantly established that IL-6 is mainly derived from the kidney contributing to high systemic levels^28^. Renal IL-1 and TNF release is a feature of I/R but not in the setting of IL-6 KO or antagonism^29^. Collectively, this could explain the discrepancy between acute phase transcriptional suppression in hepatic stress and I/R.

The approach of gene delivery of a monogenic inhibitor is not confined to IL-6 and can be exploited to target other cytokines that signal through heteromeric cytokine receptors. For instance, through replacement of the IL-6R*α* moiety by the IL-11R*α*, a mIL-11-RFP-Fc can be generated that is expected to potently block IL-11, another cytokine of the IL-6 family^6^. IL-11 has been recently identified as a dominant cytokine during gastrointestinal tumorigenesis^44^.

Taken together, to our knowledge this is the first report on the successful gene delivery of an IL-6 inhibitor that is encoded by a single gene. Very recently, the well-established anti-IL-6-R*α* antibody tocilizumab has been demonstrated to be effectively deliverable by a gene therapeutic approach^45^. However this antibody required two separate plasmids, given the nature of antibody assembly from two chains. This is simplified by the approach presented herein. IL-6 antagonism, as pioneered by the clinical use of tocilizumab and siltuximab^46^ is a promising approach for the treatment of a variety of autoimmune disorders. In our view, gene therapeutic treatment options for IL-6 antagonism that are effective and economic may be of substantial benefit for patients and society in the future.

## Methods

### Cytokines and antibodies

Murine IL-6 was purchased from ImmunoTools (Friesoythe, Germany). Anti-pY705-STAT3 (#9131), anti-STAT3 (#9139), anti-pERK1/2 (#4370), anti-ERK1/2 (#9102) from Cell Signaling (Danvers, MA), anti-V5 (#R960-25) from Invitrogen (Carlsbad, CA), anti-STAT3 C-20, anti-STAT3 K-15 (#sc-482, #sc-483), anti-ERK1 (#sc-093-G), anti-ERK2 (#sc-154-G), anti-GAPDH (#sc-32233) from Santa Cruz (Dallas, TX), anti-HSP70 (#ADI-SPA-820) from Enzo Life Sciences (Lörrach, Germany), and anti-HA (#H6908) from Sigma-Aldrich (St. Louis, MO) were used for immunoblotting. Anti-rabbit, anti-mouse and anti-goat antibodies conjugated to horseradish peroxidase were ordered from DAKO (Hamburg, Germany).

### Recombinant plasmids

mIL-6-RFP was fused to the Fc region of an engineered mIgG2a (pFuse-mIgG2ae1-Fc, Invivogen, San Diego, CA) and 3V5-3HA tag (kindly provided by Dr. Marcus Moeller, Department of Nephrology and Immunology, RWTH Aachen University, Germany) into pcDNA3.1 vector. The cDNA encoding mIL-6-RFP-Fc-3V5-3HA was optimized for murine codon usage using the GeneOptimizer algorithms (GeneArt, Regensburg, Germany). A transfer vector pMA-mIL-6-RFP-Fc-3V5-3HA containing the optimized RFP was customly synthesized (GeneArt). The cDNA for the optimized mIL-6-RFP-Fc-3V5-3HA was transferred through direct restriction cloning to generate the expression vectors pcDNA3-mIL-6-RFP-Fc-3V5-3HA (HindIII/NotI), pcDNA5/FRT/TO-mIL-6-RFP-Fc-3V5-3HA (HindIII/NotI) and PTZ-PEPCK-b.glob.intron-mIL-6-RFP-Fc-3V5-3HA-b.glob.polyA (XhoI/XhoI)^24^.

### Expression of mIL-6-RFP-Fc *in vitro*

Murine hepatocarcinoma cells (kindly provided by Dr. Christian Liedtke, Department of Medicine III, RWTH Aachen University) were cultivated in Dulbecco’s Modified Eagle Medium (DMEM) with GlutaMax™ (Invitrogen) supplemented with 10% FCS (Lonza, Basel, Switzerland), 100 U/ml penicillin and 100 μg/ml streptomycin (Sigma-Aldrich). For the cultivation of HepG2 cells (ATCC HB-8065) DMEM/F12 (Invitrogen) was used. The cells were incubated at 37°C in a water-saturated atmosphere at 5% CO_2_ and grown on 6-well plates to 70% confluence. All cells used in this study were free of mycoplasma as determined by a PCR-based assay. Cells were transfected using TransIT-LT1^®^ (Mirus Bio, Madison, WI) according to manufacturer’s instructions. Medium was exchanged to DMEM with GlutaMax™ without FCS after 4 h. For analysis of protein secretion conditioned media were harvested at the indicated time points and cleared by centrifugation.

### Protein production and purification

Stable HEK293 Flp-In/T-Rex cells (Invitrogen) for the inducible production of mIL-6-RFP-Fc were generated with the Flp-In system using 250 *μ*g/ml hygromycin B (Invivogen) and 15 *μ*g/ml blasticidin (Invivogen). Protein expression was induced in serum-free medium with 400 ng/ml doxycycline (Sigma-Aldrich) for 72 h. Harvested conditioned media were cleared through centrifugation, passed through a 0.20 *μ*m sterile-filter and afterwards loaded onto an ÄKTA Purifier 10 system (GE Healthcare, Chalfont St. Giles, UK) for affinity purification on a 1 ml Protein A Sepharose Column (#89924, Thermo Fischer, Waltham, MA). The column was washed with 20 mM sodium phosphate buffer (pH 7). mIL-6-RFP-Fc was eluted in fractions of 1 ml using 12.5 mM citric acid (pH 2.7) and neutralized immediately by adding 50 *μ*l of 2 M Tris/HCl (pH 8). Protein containing fractions were quantified using a BCA colorimetric assay (#500-0006, Bio-Rad, Munich, Germany) following manufacturer’s instruction. Protein containing fractions were pooled and dialyzed against PBS.

### Quantification of mIL-6-RFP-Fc

ELISA. Microtiter plates (Nunc Maxisorp, Sigma) were coated overnight with anti-HA antibody (#MMS-101R, BioLegend, Dedham, MA) in sterile filtered PBS at 4 °C. After blocking the plate for 1 h with 1% BSA/PBS diluted standard and samples were added and incubated for 2 h at room temperature. mIL-6-RFP-Fc bound to the plate was detected by anti-V5-HRP (#R961-25, Invitrogen). The enzymatic reaction was performed with 3,3′,5,5′-tetramethylbenzidine (TMB) substrate, stopped with 2 M H_2_SO_4_ and the absorbance was measured at 450 nm on an EON Microplate Spectrophotometer (BioTek, Winooski, VT) and analyzed with Gen5 Software (BioTek). Densitometric quantification of Western blot bands was performed with the Fiji (ImageJ) software using bands from protein standards of known concentrations.

### Inhibition of IL-6 *in vitro*

muHepa cells were grown on 6 cm dishes to 70% confluence. A mixture of IL-6 (20 ng/ml) and mIL-6-RFP-Fc (corresponding molar ratios) or PBS, respectively, was preincubated in 1 ml DMEM for 30 min and afterwards added to the cells for 20 min. For pretreatment the medium was supplemented with 1335 ng/ml mIL-6-RFP-Fc (10-fold molar excess) 90′ prior stimulation with IL-6 (20 ng/ml). Subsequently, cells were washed with PBS (137 mM NaCl, 2.5 mM KCl, 8 mM Na_2_HPO_4_, 1.5 mM KH_2_PO_4_, adjusted to pH 7.4) once and lysed with RIPA lysis buffer (50 mM Tris-HCl, pH 7.4, 150 mM NaCl, 1 mM EDTA, 0.5% Nonidet P-40, 1 mM NaF, 15% glycerol, 1 mM Na_3_VO_4_, 0.25 mM phenylmethylsulfonylfluoride (PMSF), 5 μg/ml aprotinin, and 2.5 μg/ml leupeptin). The proteins were separated by SDS-PAGE and transferred to a PVDF membrane with subsequent immunodetection using specific antibodies. Primary and HRP-conjugated secondary antibodies were diluted in TBS-N buffer (20 mM Tris-HCl, pH 7.5, 135 mM NaCl, 0.1% Nonidet P-40). Membrane-bound antibody complexes were detected by chemiluminescence (pCA-ECL solution (100 mM Tris-HCl pH 8.8, 2.5 mM luminol, 0.2 mM para coumaric acid, 2.6 mM hydrogenperoxide))^47^ using a digital imaging system (LAS 4000 mini, Fuji, Japan).

The murine pre-B cell line Ba/F3 was stably transfected through electroporation with an expression vector encoding murine gp130. Ba/F3-mgp130 cell pools expressing mgp130 in conjunction with the neomycin resistance gene were selected and maintained in the presence of 1.5 mg/ml neomycin (Invivogen) and IL-3. Ba/F3-mgp130 cells were retrovirally transduced with mIL-6R*α* using the pMOWS system as described elsewhere^48^, selected and maintained in the presence of 1.5 *μ*g/ml puromycin and 10 ng/ml IL-6. Ba/F3 cell lines were cultured in DMEM containing 10% FCS, 100 U/ml penicillin and 100 U/ml streptomycin at 37°C with 5% CO_2_ in a water-saturated atmosphere. For the proliferation assay Ba/F3-mgp130-mIL6R*α* were seeded on 96-well plates (20,000 cells/well), and stimulated with IL-6 (37 pM) in the presence of either mIL-6-RFP-Fc- or mIL-6-RFP-conditioned media. After 60 h of incubation, metabolically active cells were quantified using the cell proliferation kit II XTT assay (Roche Diagnostics, Mannheim, Germany).

### Animals and experimental design

All animal experiments have been approved by the local government authorities (Landesamt für Natur, Umwelt und Verbraucherschutz NRW (LaNUV), Düsseldorf, Germany) and were performed in accordance with relevant guidelines and regulations. Mice were held under a 12-hour light-dark cycle and had *ad libitum* access to drinking water and standard chow. Treatment groups were distributed equally among cages. For assessment of pharmacokinetics and for IL-6 injection experiments, matched male littermates aged 12–20 weeks were used. Volumes of 100 and 200 *μ*l were administered for i.v. and i.p. injections, respectively. Blood draws and euthanasia for organ harvesting were performed at the time points indicated. For subsequent quantitative protein analysis samples for comparison were assayed on a single membrane or ELISA plate with equal amounts loaded and the experimenter having animal numbers rather than specified treatment group information. qPCR, serum creatinine measurements and tissue analysis were conducted in a completely blinded fashion. Serum creatinine was analyzed as described^21^.

### Hydrodynamics-based *in vivo* gene delivery

To obtain maximal expression levels, younger, i.e., 7 week-old male littermates were transfected *via* tail vein injection of plasmid PTZ-PEPCK-b.glob.intron-mIL-6-RFP-Fc-3V5-3HA-b.glob.polyA prepared in delivery solution (TransIT-EE, Mirus Bio, Madison, WI).

### Renal ischemia and reperfusion procedure

Seven-week-old male C57/Bl6J animals (purchased from Charles River, Sulzfeld, Germany; allowed 1 week of acclimatization) hydrodynamically pre-transfected and exhibiting mIL-6-RFP-Fc levels of 0.25–4.5 *μ*g/ml (as assessed by serum ELISA at 20 h post transfection) or having received 100% of the control plasmid volume i.v. were subjected to I/R. Mice were anaesthetized using Ketamin/Xylazin, acclimatized on a warmed electrical plate for 15 min (constant plate temperature of 37 °C), and subject to operation by midline laparotomy and subsequent clamping of both renal hila using mini clamps (#18055-04, Fine Science Tools, Heidelberg). Clamping was timed to be exactly 33 min per each kidney while animals were held at a constant plate temperature of 37 °C. Completeness of ischemia of both kidney was assured by color changes of the entire organ to a dark and lighter color during and following ischemia, respectively. Mice were sutured using standard techniques. Surgeons were blinded to the animals’ treatment groups. Upon euthanasia at sacrifice animals were perfused intraarterially with saline 0.9% prior to organ harvest.

### Immunofluorescence

Prior to liver harvesting by snap-freezing (TissueTek, Sakura, Japan) mice were perfused intraarterially with saline 0.9% upon euthanasia to minimize blood cell autofluorescence. Cryosections (5 μm) were fixed in 4% paraformaldehyde for 15 min, blocked with 10% donkey serum in PBS, and subsequently stained with rabbit anti-HA antibody (1:100), donkey anti-rabbit-cy3 (1:200, Dianova, Hamburg, Germany), and DAPI (4′,6-diamidino-2-phenylindole, 1:10000, Roche, Mannheim, Germany). GFP fluorescence was preserved during this procedure. A BZ-9000 microscope (Keyence, Japan) was used for visualization.

### Histology and renal tissue injury score

Following harvest, right kidney halves were fixed in methyl Carnoy’s solution, embedded in paraffin, sectioned (2 μm), and stained by periodic acid-Schiff (PAS) as described^21^. Left kidney tissue was not available for PAS stain due to processing for cryopreservation. Renal tissue injury was assessed in a blinded fashion by scoring the percentage of tubules in the outer medulla that exhibited tubular dilation/atrophy, cast formation, cellular necrosis and loss of brush border as follows: 0, none; 1, >0–25%; 2, 25–50%, 3, 50–75% and 4, >75% as described^49^. Six consecutive non-overlapping high-power fields (×200) per section were examined.

### Preparation of liver and kidney lysates

Tissue protein extracts were generated on ice using RIPA buffer and processed for Western blot analysis similar to cellular lysates (see above). Equal amounts of protein (30 μg) were loaded.

### Quantitative mRNA analysis

RNA isolation and cDNA synthesis from tissue were performed using standard columns (Qiagen, Hilden, Germany) and random primers (Roche), respectively. Real-time quantitative PCR was carried out using qPCR Core Kit for SYBR Green (Eurogentec, Liege, Belgium) and an ABI Prism 7300 sequence detector (Life Technologies, Carlsbad, CA). Data were normalized using glyceraldehyde-3-phosphate dehydrogenase *(Gapdh)* as an internal control and calculated using the ΔΔCT-method. The following primer sequences were used: *Gapdh*, *serum amyloid A1 (Saa1)*, and *a2-macroglobulin (A2m)* as described elsewhere^21^, *Il6* fwd 5′-TGT TCA TAC AAT CAG AAT TGC CAT T-3′ and rev 5′-AGT CGG AGG CTT AAT TAC ACA TGT T-3′, *Neutrophil gelatinase-associated lipocalin (NGAL)/lipocalin-2 (Lcn2)* fwd 5′-GGC CTC AAG GAC GAC AAC A-3′ rev 5′-TCA CCA CCC ATT CAG TTG TCA-3′.

### Statistical analysis

Unpaired one-sided student’s t-test with Welch’s correction when appropriate was performed at all instances when not otherwise specified to compare control- and RFP-treated groups as indicated using GraphPad Prism version 6.0b for Mac OS X, GraphPad Software (La Jolla, CA). The one-sided test approach was chosen since our hypothesis H1 was one-sided, i.e., mIL6-RFP-Fc inhibits IL-6 or is beneficial in the renal ischemia-reperfusion model compared to control.

## Additional Information

**How to cite this article**: Görtz, D. *et al.* Anti-interleukin-6 therapy through application of a monogenic protein inhibitor *via* gene delivery. *Sci. Rep.* **5**, 14685; doi: 10.1038/srep14685 (2015).
